# Integrin-Dependent Transient Density Increase in Detergent-Resistant Membrane Rafts in Platelets Activated by Thrombin

**DOI:** 10.3390/biomedicines12010069

**Published:** 2023-12-27

**Authors:** Keisuke Komatsuya, Masaki Ishikawa, Norihito Kikuchi, Tetsuya Hirabayashi, Ryo Taguchi, Naomasa Yamamoto, Morio Arai, Kohji Kasahara

**Affiliations:** 1 Biomembrane Group, Tokyo Metropolitan Institute of Medical Science, Tokyo 156-8506, Japan; komatsuya-ks@igakuken.or.jp (K.K.); kikuchi-nr@igakuken.or.jp (N.K.); hirabayashi-tt@igakuken.or.jp (T.H.); naomasayakoby@gmail.com (N.Y.);; 2 Laboratory of Clinical Omics Research, Department of Applied Genomics, Kazusa DNA Research Institute, Kisarazu, Chiba 292-0818, Japan; mishika@kazusa.or.jp; 3 Department of Metabolome, Graduate School of Medicine, The University of Tokyo, 7-3-1 Hongo, Bunkyo-ku, Tokyo 113-0033, Japan; 4 Sado General Hospital, Niigata 952-1209, Japan

**Keywords:** lipid rafts, detergent-resistant membrane, sucrose density gradient, phosphatidylserine, platelets, clot retraction, Glanzmann’s thrombasthenia, integrin αIIbβ3, fibrin, myosin

## Abstract

Platelet lipid rafts are critical membrane domains for adhesion, aggregation, and clot retraction. Lipid rafts are isolated as a detergent-resistant membrane fraction via sucrose density gradient centrifugation. The platelet detergent-resistant membrane shifted to a higher density on the sucrose density gradient upon thrombin stimulation. The shift peaked at 1 min and returned to the control level at 60 min. During this time, platelets underwent clot retraction and spreading on a fibronectin-coated glass strip. Thrombin induced the transient tyrosine phosphorylation of several proteins in the detergent-resistant membrane raft fraction and the transient translocation of fibrin and myosin to the detergent-resistant membrane raft fraction. The level of phosphatidylserine (36:1) was increased and the level of phosphatidylserine (38:4) was decreased in the detergent-resistant membrane raft fraction via the thrombin stimulation. Furthermore, Glanzmann’s thrombasthenia integrin αIIbβ3-deficient platelets underwent no detergent-resistant membrane shift to a higher density upon thrombin stimulation. As the phosphorylation of the myosin regulatory light chain on Ser19 was at a high level in Glanzmann’s thrombasthenia resting platelets, thrombin caused no further phosphorylation of the myosin regulatory light chain on Ser19 or clot retraction. These observations suggest that the fibrin–integrin αIIbβ3–myosin axis and compositional change of phosphatidylserine species may be required for the platelet detergent-resistant membrane shift to a higher density upon stimulation with thrombin.

## 1. Introduction

Lipid rafts are dynamic assemblies of glycosphingolipids, sphingomyelin, cholesterol, and proteins that can be stabilized into platforms involved in the regulation of various vital cellular processes [1]. Rafts at the cell surface may play an important role in signal transduction. A number of studies provide considerable evidence that rafts are integral to the regulation of immune and neuronal signaling. Lipid rafts are also involved in hemostasis and thrombosis [2]. Among blood cells, platelets are critical for maintaining the integrity of the blood coagulation system. Platelet rafts are critical membrane domains in physiological responses such as adhesion, aggregation, and clot retraction [3,4]. Lipid rafts are isolated as a detergent-resistant membrane (DRM) fraction via sucrose density gradient centrifugation [5]. Platelet DRM was shown to be rich in the glycoprotein CD36 and Lyn [6,7].

We investigated the signaling in lipid rafts of neurons and platelets [8,9,10,11,12,13,14,15,16]. We demonstrated that a monoclonal antibody to ganglioside GD3, R24, co-immunoprecipitates GPI-anchored adhesion molecule TAG-1, src-family tyrosine kinase Lyn, and transmembrane raft protein Cbp from Triton X-100 extracts of rat primary cerebellar granule cells in vitro [9,10,12]. TAG-1, Lyn, Cbp, and GD3 are detected in DRM raft fraction of cerebellar granule cells [9,10,12]. These observations suggest that there is a possible association of TAG-1, Lyn, and Cbp with GD3 on the cerebellar granule cell membrane in vivo. Binding of R24 to GD3 activated Lyn and induced rapid tyrosine phosphorylation of Cbp in intact rat primary cerebellar granule cells [9]. This suggests that the GD3–Lyn association is not an artifact of detergent extraction and that GD3 can mediate transmembrane signaling to Cbp in cerebellar granule cells via Lyn [8]. Furthermore, we reported that ligation of TAG-1 induces Lyn activation and phosphorylation of Cbp and regulates migration of cerebellar granule cells during early stage of development [15]. Single-molecule imaging techniques used by Suzuki and Kusumi proved that the ligand-induced GPI-anchored protein cluster rafts recruit ganglioside and the Src-family kinases and trigger downstream signaling in intact cells [17]. Therefore, characterization of DRM is important for studying transmembrane signaling via lipid rafts.

Previously, we demonstrated that fibrin translocation to platelet DRM rafts is a specific event in platelet activation by thrombin, and clot retraction is mediated by the coagulation factor XIII-dependent fibrin–αIIbβ3–myosin axis in platelet sphingomyelin-rich rafts [13]. In this study, we demonstrated that rapid and transient platelet DRM shifts to a higher density in sucrose density gradients upon thrombin stimulation.

## 2. Materials and Methods

### 2.1. Materials

The anti-human platelet myosin polyclonal antibody (BT-561), anti-His-tag polyclonal antibody (PM002), and anti-phosphotyrosine monoclonal antibody (PY20) used were obtained from Biomedical Technologies, Medical & Biological Laboratories, and BD Transduction, respectively. Anti-phospho-Myosin Light Chain 2 (Ser19) monoclonal antibody (#3675), anti-phospho-p44/42 MAPK (Thr202/Tyr204) polyclonal antibody (#9101), and anti-p44/42 MAPK polyclonal antibody (#9102) were obtained from Cell Signaling Technology. The anti-integrin αIIb polyclonal antibody (H-160) sc-15328 and anti-phospho-integrin β3 (Tyr759) sc-20235R were purchased from Santa Cruz Biotechnology. Anti-Myosin Light Chain 2 antibody (GXT102091) and anti-Lyn monoclonal antibody (Lyn8) were obtained from Gene Tex, Wako Chemicals, respectively. Alexa Fluor 488 anti-rabbit IgG was purchased from Molecular Probes. Fibronectin (341635), thrombin (T7513), and horm collagen were obtained from Calbiochem, Sigma-Aldrich (St. Louis, MO, USA) and Nycomed Pharma (Zürich, Switzerland), respectively. Eptifibatide was obtained from LKT Laboratories (St. Paul, MN, USA).

### 2.2. Patient

A 52-year–old female patient was diagnosed as having Glanzmann’s thrombasthenia. The study was approved by the ethics committee of the Tokyo Metropolitan Institute of Medical Science 21-11. The patient gave informed consent in accordance with the Declaration of Helsinki. Informed consent was obtained from all subjects involved in the study.

### 2.3. Platelet Preparation

Blood was collected into 3.8% sodium citrate at a ratio of 9:1. The blood was centrifuged (140× *g*) to prepare platelet-rich plasma (PRP). To prepare washed platelets, PRP was incubated with 4 mM citric acid and washed with Tyrode’s buffer (137 mM NaCl, 2.7 mM KCl, 3.75 mM NaH_2_PO_4_, 5 mM HEPES, 0.35% BSA, and 5 mM glucose; pH 6.8) containing 1 mM PGE1 and 1 U/mL heparin. Finally, the platelets were resuspended in Tyrode’s buffer (137 mM NaCl, 2.7 mM KCl, 3.75 mM NaH_2_PO_4_, 5 mM HEPES, 0.35% BSA, and 5 mM glucose; pH 7.4) containing 1 mM CaCl_2_ and 1 mM MgCl_2_.

### 2.4. Sucrose Density Gradient Analysis

Platelets (6 × 10^9^) were homogenized using a Teflon glass homogenizer in 2 mL of TNE/Triton buffer (0.05% Triton X-100, 25 mM Tris-HCl, pH 7.5, 150 mM NaCl, and 1 mM EGTA). The sucrose content was then adjusted to 40% by adding 80% sucrose. A sucrose gradient (5–30%) in 6 mL of TNE without Triton X-100 was layered over the lysate, and the contents were centrifuged for 17 h at 39,000 rpm at 4 °C in a Hitachi RPS40T rotor (Tokyo, Japan). The ten fractions were collected from the top of the gradient. Small-scale method was used for time-course and Glanzmann’s thrombasthenia platelets analysis. Platelets (6 × 10^8^) were homogenized using a Teflon glass homogenizer in 100 μL of TNE/Triton buffer (0.05% Triton X-100, 25 mM Tris-HCl, pH 7.5, 150 mM NaCl, and 1 mM EGTA). The sucrose content of the homogenate was then adjusted to 40% by adding 80% sucrose, placed at the bottom of an ultracentrifuge tube (0.9 PC Thick-walled tube, Hitachi S304296A), and overlaid with 35% and then 5% sucrose in 300 μL of TNE without Triton X-100. The discontinuous gradient was centrifuged for 17 h at 35,000 rpm at 4 °C in a Hitachi RPS40T rotor with 2S13 adaptor (Hitachi, 336698A).

### 2.5. Clot Retraction

PRP was activated with 1 U/mL thrombin and 5 mM CaCl_2_, and the reaction mixtures were left unstirred at 37 °C in siliconized tubes. The extent of clot retraction was monitored by taking photographic images [17].

### 2.6. Cell Immunostaining

To stain integrin β3 on the platelet surface, platelets were adhered onto a glass-bottomed culture dish coated with 100 μg/mL fibronectin using 1 U/mL thrombin, fixed in 4% paraformaldehyde, and incubated with anti-integrin β3 monoclonal antibody TM83 [18] for 1 h, and then with the Alexa Fluor 488-labeled secondary antibody. The images were captured using a Carl Zeiss confocal imaging system (LSM510 META). The time-lapse platelet spreading differential interference contrast (DIC) images were captured using an Olympus LCV110.

### 2.7. Binding Assay of Fibrinogen γ Chain C-Terminal Fusion Protein

The fibrinogen γ chain C-terminal (residues 144-411) was expressed with HEK293T cells as a fusion protein, with an N-terminal human growth hormone domain, using the pSGHV0 vectors; gifts from Dr. J. Takagi [19]. The linker between the fibrinogen γ-chain and the growth hormone contains a His8 tag. The fusion protein was purified with Ni-NTA chromatography. A mixture of 300,000/μL washed platelets, 12 μg/mL fibrinogen γ chain C-terminal fusion protein, 1 mM CaCl_2_, and 1 mM MgCl_2_ in HEPES-Tyrode’s buffer was stimulated with 0.5 U/mL thrombin for 15 min and centrifuged. After the removal of the supernatant, i.e., the unbound fraction, the precipitate was subjected to sucrose density gradient analysis.

### 2.8. Lipid Extraction

The platelet samples were homogenized with chloroform/methanol (1:2, *v*/*v*). Phospholipids (PLs) were extracted using the Bligh and Dyer method [20]. The total lipid extract was dried under a gentle stream of nitrogen, dissolved in 10 mL of chloroform/methanol (1:1, *v*/*v*) and stored at −20 °C.

### 2.9. Mass Spectrometry

The targeted lipidomics was performed via direct infusion at a flow rate of 15 µL/min. Mobile phase consisted of methanol/acetonitrile/H_2_O (19/19/2) containing 0.1% acetic acid and ammonium hydroxylate (28%). Phospholipids were analyzed with precursor ion scanning and neutral loss scanning of polar head groups [21] using a TSQ Vantage triple-quadrupole mass spectrometer (ThermoElectron Corp., San Jose, CA, USA), coupled with a LC-10ADVPµ HPLC system (Shimadzu, Kyoto, Japan). Phosphatidylserines (PSs) and Phosphatidylethanolamines (PEs) were measured through neutral loss scanning of polar head groups (PS, 185.1 amu; PE 141.1 amu) in the positive ion mode. Phosphatidylcholines (PCs) were measured with precursor ion scanning of polar head groups-related fragment ion (*m*/*z* 184) in the positive ion mode. Relative quantification of PLs were calculated via intensity ratio (compared to molecule with highest intensity).

## 3. Results and Discussion

### 3.1. Transient Platelet DRM Shifted to a Higher Density upon Thrombin Stimulation

**Figure 1:**
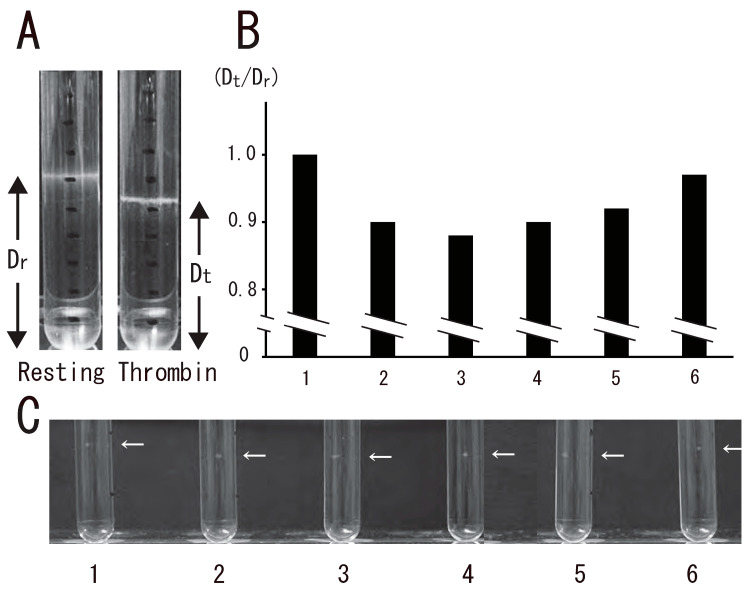
Transient platelet DRM shifted to a higher density by thrombin stimulation. (**A**) Photo images of DRM in sucrose density gradient analysis of resting platelets (**left photo**) and platelets stimulated for 3 min with 1 U/mL thrombin (**right photo**). DRM positions in the sucrose density gradient were shown. Dr, distance from the bottom of the gradient in resting platelets; Dt, distance from the bottom of the gradient in thrombin-stimulated platelets for the indicated times. (**B**) Time-dependent DRM shift in sucrose density gradient analysis shown by the ratio between Dt and Dr (Dt/Dr). Lane 1, 0 min; lane 2, 0.5 min; lane 3, 1 min; lane 4, 5 min; lane 5, 15 min; lane 6, 60 min. (**C**) Photo image of time-dependent DRM shift using small-scale method. Arrows indicate the position of DRM. Lane 1, 0 min; lane 2, 0.5 min; lane 3, 1 min; lane 4, 5 min; lane 5, 15 min; lane 6, 60 min.

**Figure 2:**
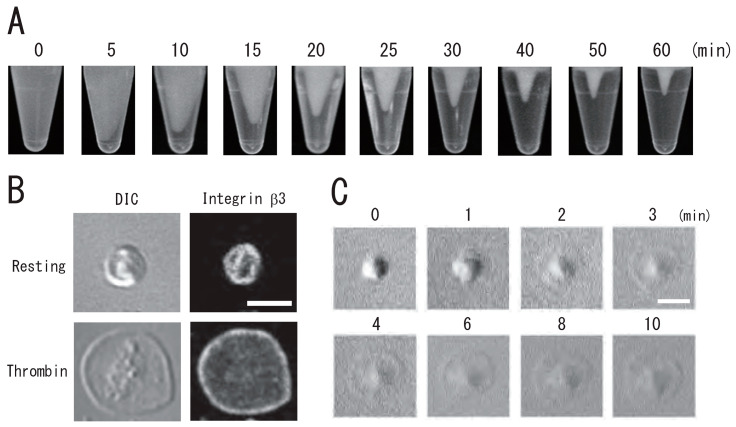
Time-lapse clot retraction and platelet spreading due to thrombin. (**A**) Time-lapse photo images of clot retraction assay for 0, 5, 10, 15, 20, 25, 30, 40, 50, and 60 min. (**B**) Immunocytochemical of integrin β3 in resting (**upper**) and spreading (**lower**) platelets. Left panels, differential interference contrast (DIC); right panels, immunostaining with anti-integrin β3 antibody. (**C**) Time-lapse DIC images of platelet spreading due to treatment with thrombin for 0, 1, 2, 3, 4, 6, 8, and 10 min. Scale bar, 3 μm.

DRM, a low-density light-scattering band, was detected through sucrose density gradient analysis of resting platelets. The platelet DRM shifted to a higher density upon treatment with thrombin for 5 min (Figure 1A). Thrombin caused the rapid DRM shift within 30 s. The shift peaked at 1 min and returned to the resting level at 60 min (Figure 1B). These observations suggested transient density increase in DRM rafts in activated platelets with thrombin. Next, we investigated the time-dependent fibrin clot retraction and platelet spreading upon thrombin stimulation. Clot retraction is mediated by the interaction of the fibrin fiber and actomyosin via the integrin αIIbβ3, together with the activation of the platelet contractile apparatus [22,23,24]. PRP was activated with thrombin, and the reaction mixtures were left unstirred at 37 °C in siliconized tubes. The extent of clot retraction was monitored by taking photographic images. Thrombin caused clot retraction within 5 min and time-dependent retraction for 1 h (Figure 2A). Platelets were adhered onto a fibronectin-coated glass strip and spread with thrombin for 15 min. DIC imaging and immunostaining with an anti-integrin β3 monoclonal antibody clearly demonstrated platelet spreading (Figure 2B). Figure 2C shows time-lapse platelet spreading induced by thrombin for 10 min.

### 3.2. Proteins and Fibrin Translocation to DRM Raft Fraction of Human Platelets by Thrombin Stimulation

**Figure 3:**
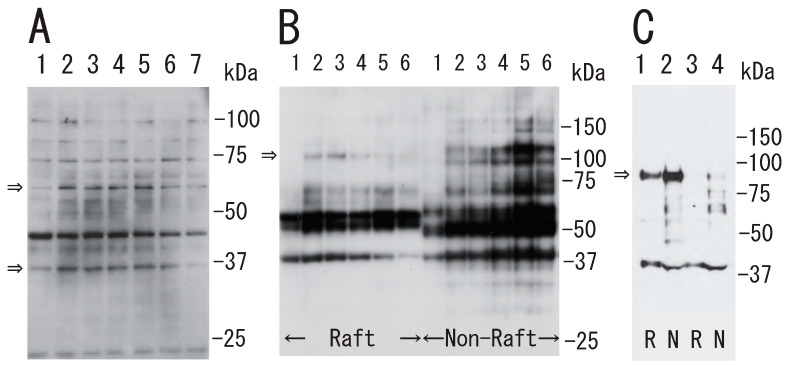
Protein tyrosine phosphorylation in platelets via thrombin stimulation. (**A**) Time-dependent tyrosine phosphorylation of the protein. After treatment of platelets with thrombin (0.5 U/mL) for the indicated times, the lysates were subjected to SDS-PAGE and immunoblotted with anti-phosphotyrosine antibody. Lane 1, 0 min; lane 2, 0.5 min; lane 3, 1 min; lane 4, 2 min; lane 5, 5 min; lane 6, 10 min; lane 7, 30 min. Arrows indicate tyrosine phosphorylation of ~60 kDa and ~37 kDa proteins. (**B**) Time-dependent tyrosine phosphorylation in DRM raft and non-raft fraction. After treatment of platelets with thrombin for the indicated times, the Triton X-100 extracts were subjected to sucrose density gradient analysis. Lane 1, 0 min; lane 2, 0.5 min; lane 3, 1 min; lane 4, 5 min; lane 5, 15 min; lane 6, 60 min. Arrow indicates tyrosine phosphorylation of ~100 kDa protein in DRM raft fraction. (**C**) Phosphorylation of integrin β3 at Tyr759 via thrombin stimulation using anti-phospho-integrin β 3 (Tyr759) antibody. Untreated resting platelets (lane 1, 2), and thrombin-treated platelets for 10 min (lane 3, 4) were subjected to sucrose density gradient analysis. Lane 1, 3, R: DRM raft fraction; lane 2, 4, N: non-raft fraction. Arrow indicates phosphorylation of integrin β3 at Tyr759.

What is the mechanism of the transient platelet DRM shift to a higher density upon stimulation with thrombin? To answer this question, we investigated transient events in the DRM of platelets with thrombin stimulation. The treatment of platelets with thrombin induced the tyrosine phosphorylation of several proteins, including 60 and 37 kDa proteins. The phosphorylation peaked at 0.5 min and returned to the control level at 30 min (Figure 3A). The transient tyrosine phosphorylation of 100 kDa protein with the peak at 1 min was detected in the DRM fraction (Figure 3B). Thrombin induced the tyrosine dephosphorylation of integrin β3 at tyrosine 759 in both raft and non-raft fractions (Figure 3C).

**Figure 4:**
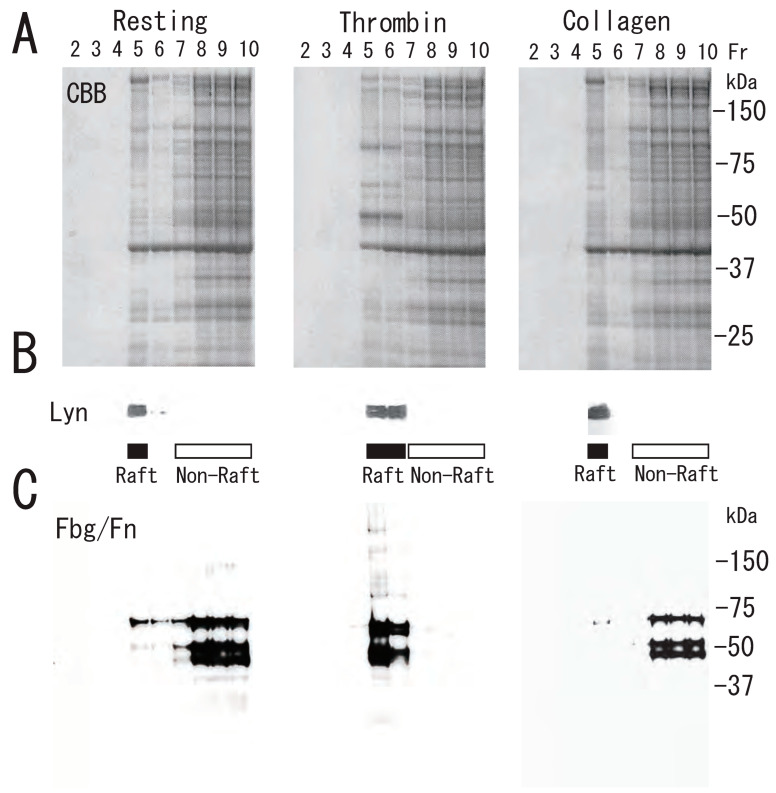
Proteins and fibrin translocation to DRM raft fraction of human platelets via thrombin, but not collagen, stimulation. (**A**) Sucrose density gradient analysis of proteins in washed human platelets. Resting platelets (**left panel**), thrombin (1 U/mL for 3 min)-stimulated platelets (**middle panel**), and collagen (10 μg/mL for 3 min)-stimulated platelets (**right panel**) were lysed in Triton X-100, and sucrose gradients (5% to 30%) were formed over them. Ten fractions were collected from top to bottom after centrifugation. The proteins were subjected to SDS-PAGE and stained with Coomassie brilliant blue. (**B**) Immunoblotting of raft marker protein Lyn with anti-Lyn antibody. (**C**) Immunoblotting with anti-fibrinogen polyclonal antibody [4].

**Figure 5:**
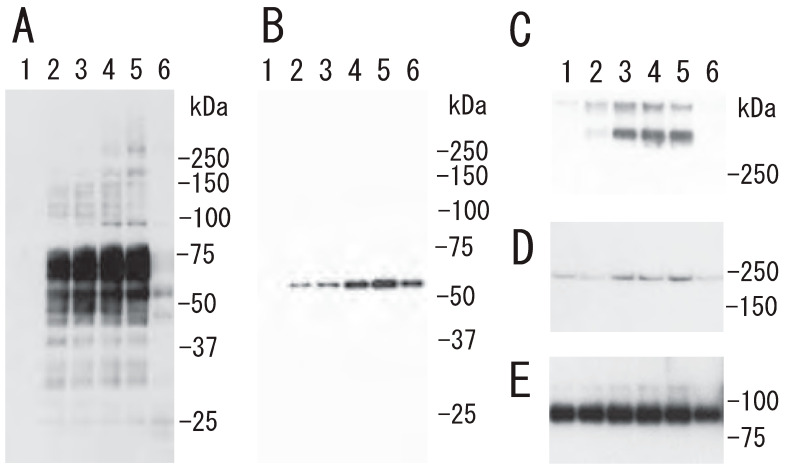
Time-dependent translocation of fibrin, fibrinogen γ-chain fusion protein, and myosin to DRM raft fraction via thrombin. A mixture of platelets and fibrinogen γ chain fusion protein was stimulated with 0.5 U/mL thrombin for 15 min and centrifuged. The precipitate was subjected to sucrose density gradient analysis. (**A**) Immunoblotting with anti-fibrinogen polyclonal antibody. (**B**) Immunoblotting of fibrinogen γ chain fusion protein with anti-His-tag polyclonal antibody. (**C**) Immunoblotting under nonreduced condition with anti-myosin antibody. (**D**) Immunoblotting under reduced condition with anti-myosin antibody. (**E**) Immunoblotting with integrin β3 antibody.

Next, we investigated the association of specific proteins with the DRM rafts of activated platelets. Several Coomassie Brilliant Blue (CBB)-stained proteins were translocated to DRM (fractions 5 and 6) via treatment with thrombin for 3 min (Figure 4A). As we previously demonstrated, one of the several CBB-stained proteins is fibrin [13]. Lyn, a lipid raft marker, was translocated from fraction 5 of resting platelets to fractions 5 and 6 of activated platelets upon thrombin stimulation (Figure 4B). These observations also suggested a rapid platelet DRM shift to a higher density upon thrombin stimulation. Immunoblotting with anti-fibrinogen antibody showed fibrin translocation to the DRM fraction of activated platelets by thrombin. In resting platelets, fibrinogens Aα (67 kDa), Bβ (52 kDa), and γ (47 kDa) were detected in the non-raft fractions (fractions 7–10). In contrast, fibrins α (65 kDa), β (50 kDa), and γ (47 kDa) were detected in the DRM fractions (fractions 5 and 6) of thrombin-stimulated platelets (Figure 4C). These findings suggest that fibrinogen is released from α-granules of platelets, converted to fibrin by the cleavage of fibrinopeptides A and B by thrombin, and translocated to lipid rafts of thrombin-stimulated platelets [4]. However, the fibrinogen translocation to the DRM fraction and platelet DRM shift to a higher density were not detected in collagen-stimulated platelets (Figure 4B,C). This different effect may be due to no conversion of fibrinogen to fibrin and no fibrin fiber formation by collagen stimulation. These observations suggested that the conversion of fibrinogen to fibrin by thrombin and further fibrin crosslinking by coagulation factor XIII are necessary for the platelet DRM shift to a higher density on a sucrose density gradient upon thrombin stimulation. To support this idea, fibrin fiber is directly associated with the surface of DRM of thrombin-stimulated platelets via immunoelectron microscopy [13]. Furthermore, the thrombin-induced fibrin-translocation to DRM fraction is impaired in coagulation factor XIII A subunit-deficient platelets [13]. The translocation of fibrin to the DRM fraction of thrombin-stimulated platelets was time-dependent (Figure 5A). Thrombin caused the rapid translocation of fibrin to DRM within 30 s. The translocation returned to the resting level at 60 min. The carboxyl termini (144-411) of the γ-chains of human fibrin provide recognition sites for the binding of the fibrin protofibril to αIIbβ3 on activated human platelets. The fibrinogen γ-chain C-terminal fusion protein transiently bound to the DRM of thrombin-stimulated platelets (Figure 5B). Thrombin also caused the rapid translocation of myosin to DRM within 1 min. The myosin translocation returned to the resting level at 60 min (Figure 5C,D). In contrast, integrin β3 was detected in the DRM raft fraction of resting platelets. The amount of integrin β3 was not changed via thrombin stimulation (Figure 5E).

Platelet DRM shifts to a higher density in sucrose gradients upon platelet activation, suggesting that platelet lipid rafts are dynamic membrane microdomains. In this study, we demonstrated that fibrin and myosin are transiently translocated to the DRM raft fractions of thrombin-stimulated platelets. The possible mechanism of the transient DRM shift to a higher density in sucrose gradients upon platelet activation via thrombin presumably involves the high protein-to-lipid ratio [25]. Consistent with this idea, two distinct types of DRM were obtained after sucrose density gradient centrifugation using Triton X-100. Light DRM contained cerebroside, whereas heavy DRM contained Ca^2+^ATPase and the IP3 receptor in porcine lung membranes [26]. Two distinct types of DRM were also obtained after sucrose density gradient centrifugation using Brij 98. Light DRM contained ganglioside GM1 and MHC II, whereas heavy DRM contained ganglioside GM2 and MHC I in B-lymphocytes [27].

### 3.3. A Change in Phospholipids Composition of DRM Raft Fraction by Thrombin Stimulation

**Figure 6:**
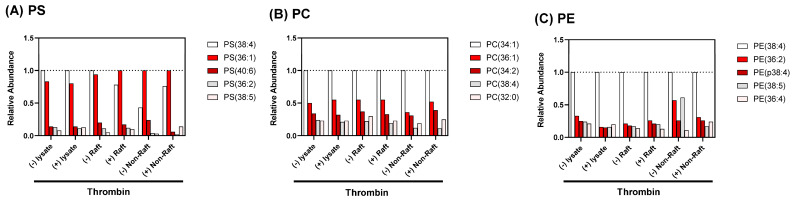
Relative abundance of phospholipid molecular species in total lysate, DRM raft fraction, and non-raft fraction of human platelets with thrombin stimulation. (**A**) Phosphatidylserines, (**B**) phosphatidylcholines, (**C**) phosphatidylethanolamines. Odd number lanes; resting platelets. Even number lanes; platelets activated via 0.2 U/mL thrombin stimulation for 10 min.

Lipid rafts are composed of lipids and proteins. Upon stimulation, platelets are known to flip phosphatidylserine (PS) from the inner monolayer to the outer monolayer, which could also change lipid rafts. Therefore, we investigated a possible global change in lipid composition of DRM raft fraction. We performed the targeted-lipidomics to obtain PLs profiles of platelet-derived samples. As a result, PS(36:1), PS(36:2), PS(38:4), PS(38:5), and PS(40:6) were identified from the lipid extracts (Figure 6A and Figure S1). Interestingly, the level of PS(36:1) in the raft fractions was increased and the level of PS(38:4) in the raft fractions was decreased by the thrombin stimulation (Figure 6A and Table 1). In contrast to the raft fractions, the level of PS(38:4) in non-raft fractions was increased by the stimulation. In the lysates, the level of PS(38:4) level were comparable between resting platelets lysate and thrombin-stimulated platelets lysate. These findings suggest that thrombin stimulation may modulate the metabolism of polyunsaturated fatty acids-containing PSs in the platelets and induce the alterations of PSs profiles in the raft membrane. In addition, our results indicated that raft fractionation is important for evaluating the stimulation-induced alterations of PSs profiles. We then investigated the alterations of PCs and PEs profiles in platelet-derived samples. In PCs, no stimulation-induced profile alterations were observed in either the lysates, raft fractions, or non-raft fractions (Figure 6B and Figure S2 and Table 2). In the non-raft fractions, the level of PE(38:5) were decreased by stimulation (Figure 6C and Figure S3 and Table 3).

We demonstrated that PS(36:1) and PS(38:4) are the two major PS of human platelets (Table 1 and Figure S1). The predominant PS in human platelets are 18:0a/18:1-PS and 18:0a/20:4-PS [28]. Platelet activation via thrombin causes the two PS externalization via Ca^2+^-activated TMEM16F’s PL-scramblase activity [28,29]. Therefore, our observations suggested that a change of the ratio between 18:0a/18:1-PS and 18:0a/20:4-PS in DRM rafts may be involved in the platelet DRM shift to a higher density on a sucrose density gradient upon thrombin stimulation.

### 3.4. Impairment of Thrombin-Induced Platelet DRM Shift to a Higher Density in Type I Glanzmann’s Thrombasthenia

**Figure 7:**
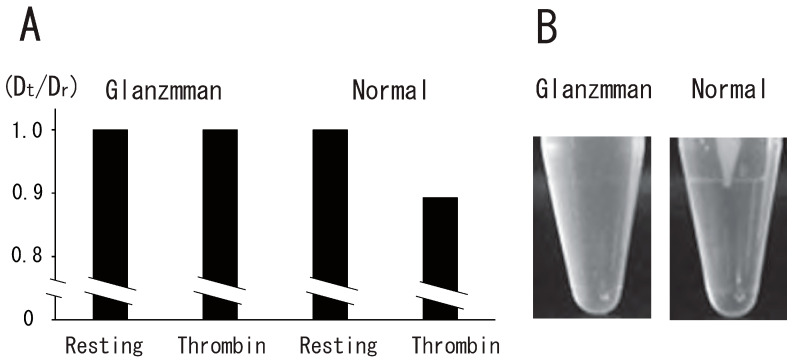
Impairment of thrombin-induced platelet DRM shift to a higher density and clot retraction in Glanzmann’s thrombasthenia. (**A**) Glanzmann’s thrombasthenia and normal platelets were treated with 1 U/mL thrombin for 5 min and were subjected to sucrose density gradient analysis. DRM shifts to a higher density were shown by the ratio between Dt and Dr (Dt/Dr). (**B**) Glanzmann’s thrombasthenia PRP (**left**) and normal PRP (**right**) were incubated with 1 U/mL thrombin and 2 mM CaCl_2_. Photo image of clot retraction assay after 60 min.

**Figure 8:**
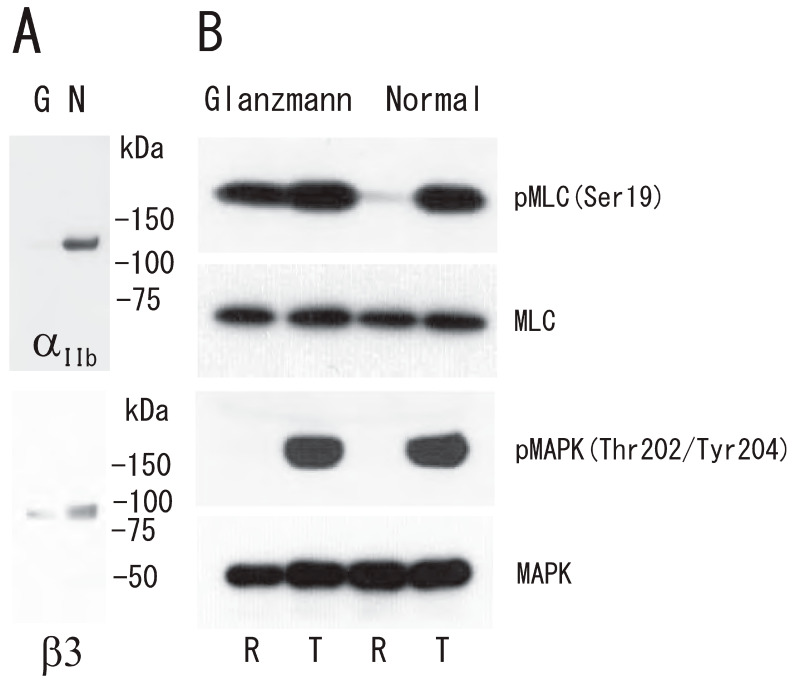
Abnormal phosphorylation of myosin light chain Ser19 in Glanzmann’s thrombasthenia platelets. (**A**) Immunoblotting with anti-integrin αIIb antibody (**upper**) and anti-integrin β3 antibody (**lower**). Lane G, Glanzmann’s thrombasthenia platelets; lane N, normal platelets. (**B**) Immunoblotting with anti-phospho-MLC (Ser19), anti-MLC, anti-phospho-p44/42 MAPK (Thr202/Tyr204), and anti-p44/42 MAPK antibody from top to bottom panel of Glanzmann’s thrombasthenia (**left**) and normal (**right**) platelets. Lane R, resting platelets; lane T, 1 U/mL thrombin-treated (5 min) platelets.

To investigate the role of integrin αIIbβ3 in the platelet DRM shift to a higher density via treatment with thrombin, we used platelets from a Glanzmann’s thrombasthenia patient, a disease characterized by the absence of αIIbβ3, a fibrin(ogen) receptor. The DRM of normal platelets, but not Glanzmann’s thrombasthenia platelets, shifted to a higher density upon treatment with thrombin for 5 min (Figure 7A). Clot retraction was also impaired in Glanzmann’s thrombasthenia platelets (Figure 7B). These observations suggest that integrin αIIbβ3 is required for clot retraction and the platelet DRM shift to a higher density upon treatment with thrombin. In platelets from a Glanzmann’s thrombasthenia patient, small amounts of integrin αIIb (4%) and integrin β3 (7%) were detected via Western blotting (Figure 8A). Next, we investigated thrombin-induced signal transduction in Glanzmann’s thrombasthenia platelets. Clot retraction is accompanied by the enhanced phosphorylation of the myosin regulatory light chain (MLC) on Ser19, which activates myosin to mediate the transmission of force to the fibrin clot during clot retraction [30,31]. Mitogen-activated protein kinase (MAPK) activation is important in facilitating clot retraction. The stimulatory role of MAPK in clot retraction is mediated by the stimulation of MLC phosphorylation [32]. In resting normal platelets, the phosphorylation of MLC Ser19 was at a low level. Thrombin stimulation caused the high phosphorylation of MLC Ser19 (Figure 8B). Interestingly, the phosphorylation of MLC Ser19 was at a high level in Glanzmann’s thrombasthenia resting platelets. Therefore, thrombin stimulation did not enhance the phosphorylation of MLC Ser19 (Figure 8B). In contrast, the phosphorylation of MAPK on Thr202/Tyr204 was at a low level in normal and Glanzmann’s thrombasthenia resting platelets. Thrombin stimulation caused the high-level phosphorylation of MAPK on Thr202/Tyr204 in normal and Glanzmann’s thrombasthenia platelets (Figure 8B).

The outside–in signaling of integrin αIIbβ3 on platelets is triggered by the binding of fibrin(ogen) [33]. The calpain cleavage of integrin β3 at Tyr759 by the dephosphorylation of Tyr759 is a molecular switch for clot retraction [34]. Myosin is involved in integrin αIIbβ3 outside–in signaling as an adaptor molecule [35]. The phosphorylation of MLC Ser19 in DRM raft fraction by thrombin was inhibited by treatment with eptifibatide, the integrin αIIbβ3 inhibitor (Figure S4). The absence of integrin αIIbβ3 in Glanzmann’s thrombasthenia platelets might mimic the absence of the molecular switch, resulting in the induction of outside–in-like signaling (RhoA–ROCK–MLC pathway) without ligand binding, leading to the phosphorylation of MLC Ser19 in resting Glanzmann’s thrombasthenia platelets. The mechanism of phosphorylation of MLC Ser19 in resting Glanzmann’s thrombasthenia platelets remains to be explored. The DRM shift to a higher density in sucrose gradients upon platelet activation was also reported by other groups [36,37]. To the best of our knowledge, this is the first report showing rapid and transient DRM shifts to a higher density in sucrose gradients upon cell activation.

## 4. Conclusions

The platelet detergent-resistant membrane shifted to a higher density on the sucrose density gradient upon thrombin stimulation. The shift peaked at 1 min and returned to the control level at 60 min. Thrombin induced the transient translocation of fibrin and myosin to the detergent-resistant membrane raft fraction. The level of PS(36:1) was increased and the level of PS(38:4) was decreased in the detergent-resistant membrane raft fraction via the thrombin stimulation. Furthermore, Glanzmann’s thrombasthenia integrin αIIbβ3-deficient platelets underwent no detergent-resistant membrane shift to a higher density upon thrombin stimulation. These observations suggest that the fibrin–integrin αIIbβ3–myosin axis and a change of the ratio between PS(36:1) and PS(38:4) in DRM rafts may be required for the platelet detergent-resistant membrane shift to a higher density upon stimulation with thrombin.

## Figures and Tables

**Table 1:** Relative abundance of PSs in the lipid extracts from platelets.

		Resting Lysate			Resting Raft			Resting Non-Raft			Thrombin Lysate			Thrombin Raft			Thrombin Non-Raft	
*m*/*z*	Species	Peak Height	Relative Abundance		Peak Height	Relative Abundance		Peak Height	Relative Abundance		Peak Height	Relative Abundance		Peak Height	Relative Abundance		Peak Height	Relative Abundance
812	PS(38:4)	4.05 × 10^5^	1.00		3.59 × 10^5^	1.00		2.80 × 10^4^	0.43		3.90 × 10^5^	1.00		3.37 × 10^5^	0.78		5.58 × 10^4^	0.76
790	PS(36:1)	3.36 × 10^5^	0.83		3.37 × 10^5^	0.94		6.50 × 10^4^	1.00		3.12 × 10^5^	0.80		4.32 × 10^5^	1.00		7.34 × 10^4^	1.00
836	PS(40:6)	5.67 × 10^4^	0.14		7.18 × 10^4^	0.20		1.56 × 10^4^	0.24		5.46 × 10^4^	0.14		5.73 × 10^4^	0.17		4.40 × 10^3^	0.06
788	PS(36:2)	5.27 × 10^4^	0.13		3.95 × 10^4^	0.11		2.60 × 10^3^	0.04		4.29 × 10^4^	0.11		4.04 × 10^4^	0.12		1.47 × 10^3^	0.02
810	PS(38:5)	3.24 × 10^4^	0.08		1.80 × 10^4^	0.05		1.95 × 10^3^	0.03		5.07 × 10^4^	0.13		3.37 × 10^4^	0.10		1.03 × 10^4^	0.14
	Total Height	8.83 × 10^5^			8.26 × 10^5^			1.13 × 10^5^			8.50 × 10^5^			9.00 × 10^5^			1.45 × 10^5^	

**Table 2:** Relative abundance of PCs in the lipid extracts from platelets.

		Resting Lysate			Resting Raft			Resting Non-Raft			Thrombin Lysate			Thrombin Raft			Thrombin Non-Raft	
*m*/*z*	Species	Peak Height	Relative Abundance		Peak Height	Relative Abundance		Peak Height	Relative Abundance		Peak Height	Relative Abundance		Peak Height	Relative Abundance		Peak Height	Relative Abundance
760	PC(34:1)	2.41 × 10^6^	1.00		3.85 × 10^6^	1.00		4.53 × 10^5^	1.00		2.14 × 10^6^	1.00		3.80 × 10^6^	1.00		2.89 × 10^5^	1.00
788	PC(36:1)	1.21 × 10^6^	0.50		2.12 × 10^6^	0.55		1.63 × 10^5^	0.36		1.18 × 10^6^	0.55		2.09 × 10^6^	0.55		1.50 × 10^5^	0.52
758	PC(34:2)	8.19 × 10^5^	0.34		1.42 × 10^6^	0.37		1.40 × 10^5^	0.31		6.85 × 10^5^	0.32		1.25 × 10^6^	0.33		1.13 × 10^5^	0.39
810	PC(38:4)	5.78 × 10^5^	0.24		8.47 × 10^5^	0.22		5.44 × 10^4^	0.12		4.49 × 10^5^	0.21		7.22 × 10^5^	0.19		3.18 × 10^4^	0.11
734	PC(32:0)	5.54 × 10^5^	0.23		1.16 × 10^6^	0.30		8.61 × 10^4^	0.19		4.92 × 10^5^	0.23		8.74 × 10^5^	0.23		7.23 × 10^4^	0.25
	Total Height	5.57 × 10^6^			9.39 × 10^6^			8.97 × 10^5^			4.94 × 10^6^			8.74 × 10^6^			6.56 × 10^5^	

**Table 3:** Relative abundance of PEs in the lipid extracts from platelets.

		Resting Lysate			Resting Raft			Resting Non-Raft			Thrombin Lysate			Thrombin Raft			Thrombin Non-Raft	
*m*/*z*	Species	Peak Height	Relative Abundance		Peak Height	Relative Abundance		Peak Height	Relative Abundance		Peak Height	Relative Abundance		Peak Height	Relative Abundance		Peak Height	Relative Abundance
768	PE(38:4)	3.32 × 10^5^	1.00		4.72 × 10^5^	1.00		2.53 × 10^4^	1.00		3.82 × 10^5^	1.00		3.47 × 10^5^	1.00		3.17 × 10^4^	1.00
744	PE(36:2)	1.10 × 10^5^	0.33		9.91 × 10^4^	0.21		1.44 × 10^4^	0.57		6.11 × 10^4^	0.16		9.02 × 10^4^	0.26		9.83 × 10^3^	0.31
752	PE(p38:4)	8.30 × 10^4^	0.25		8.50 × 10^4^	0.18		6.58 × 10^3^	0.26		5.73 × 10^4^	0.15		7.29 × 10^4^	0.21		8.24 × 10^3^	0.26
766	PE(38:5)	7.97 × 10^4^	0.24		8.02 × 10^4^	0.17		1.54 × 10^4^	0.61		6.11 × 10^4^	0.16		6.94 × 10^4^	0.20		5.39 × 10^3^	0.17
740	PE(36:4)	6.97 × 10^4^	0.21		6.61 × 10^4^	0.14		2.78 × 10^3^	0.11		7.64 × 10^4^	0.20		4.51 × 10^4^	0.13		7.61 × 10^3^	0.24
	Total Height	6.74 × 10^5^			8.02 × 10^5^			6.45 × 10^4^			6.38 × 10^5^			6.25 × 10^5^			6.28 × 10^4^	

PE(p38:4): alkenyl/acyl type PE(38:4) or alkylacyl type PE(38:5).

## Data Availability

Data are contained within the article and supplementary materials.

## Supplementary Materials

The following supporting information can be downloaded at: https://www.mdpi.com/article/10.3390/biomedicines12010069/s1.

## Abbreviations

DRM	detergent-resistant membrane
DIC	differential interference contrast
PRP	platelet-rich plasma
CBB	Coomassie Brilliant Blue
MLC	myosin regulatory light chain
MAPK	mitogen-activated protein kinase
PC	Phosphatidylcholine
PE	phosphatidylethanolamine
PL	phospholipid
PS	phosphatidylserine
Ser	serine
Thr	threonine
Tyr	tyrosine
