# Supplementary material for: Integrin-Dependent Transient Density Increase in Detergent-Resistant Membrane Rafts in Platelets Activated by Thrombin
Source: Biomedicines. 2023 Dec 27;12(1):69. doi: 10.3390/biomedicines12010069 (PMC10813660; doi:10.3390/biomedicines12010069)
Supplement: Supplementary file 1 [file biomedicines-12-00069-s001.zip › biomedicines-2721851-supplementary.pdf]

Figure S1. Mass spectra of PSs

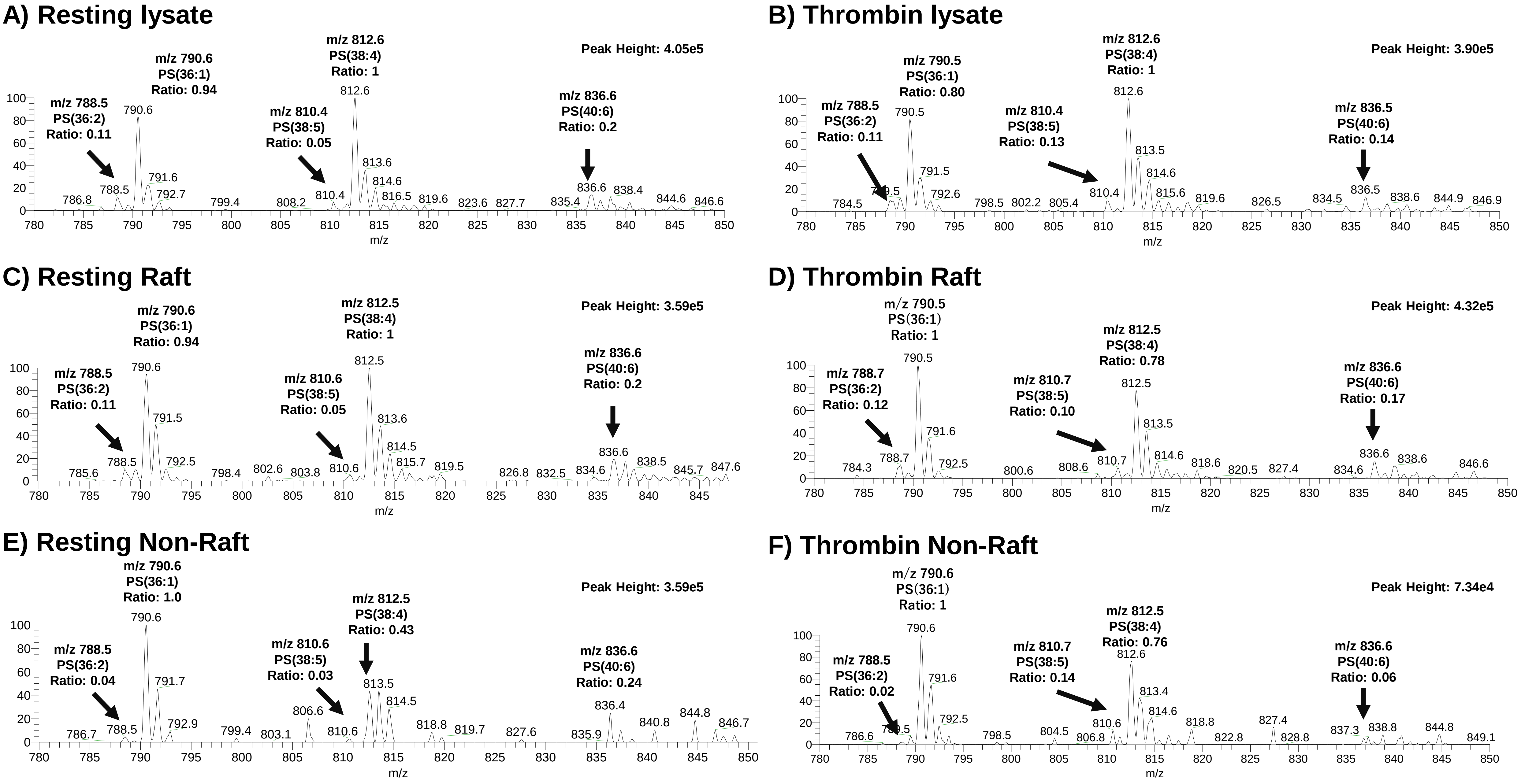

Figure S2. Mass spectra of PCs

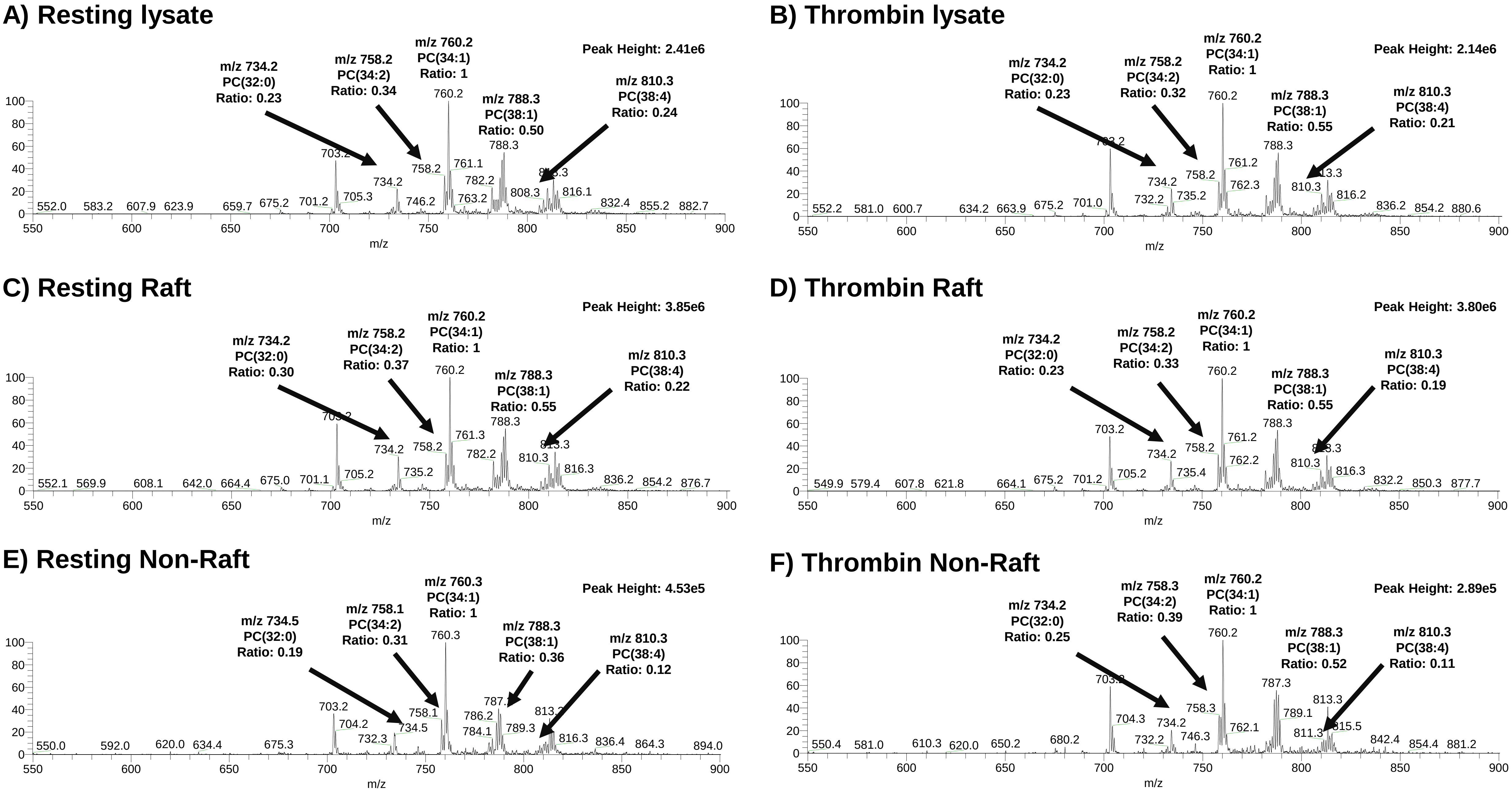

Figure S3. Mass spectra of PEs

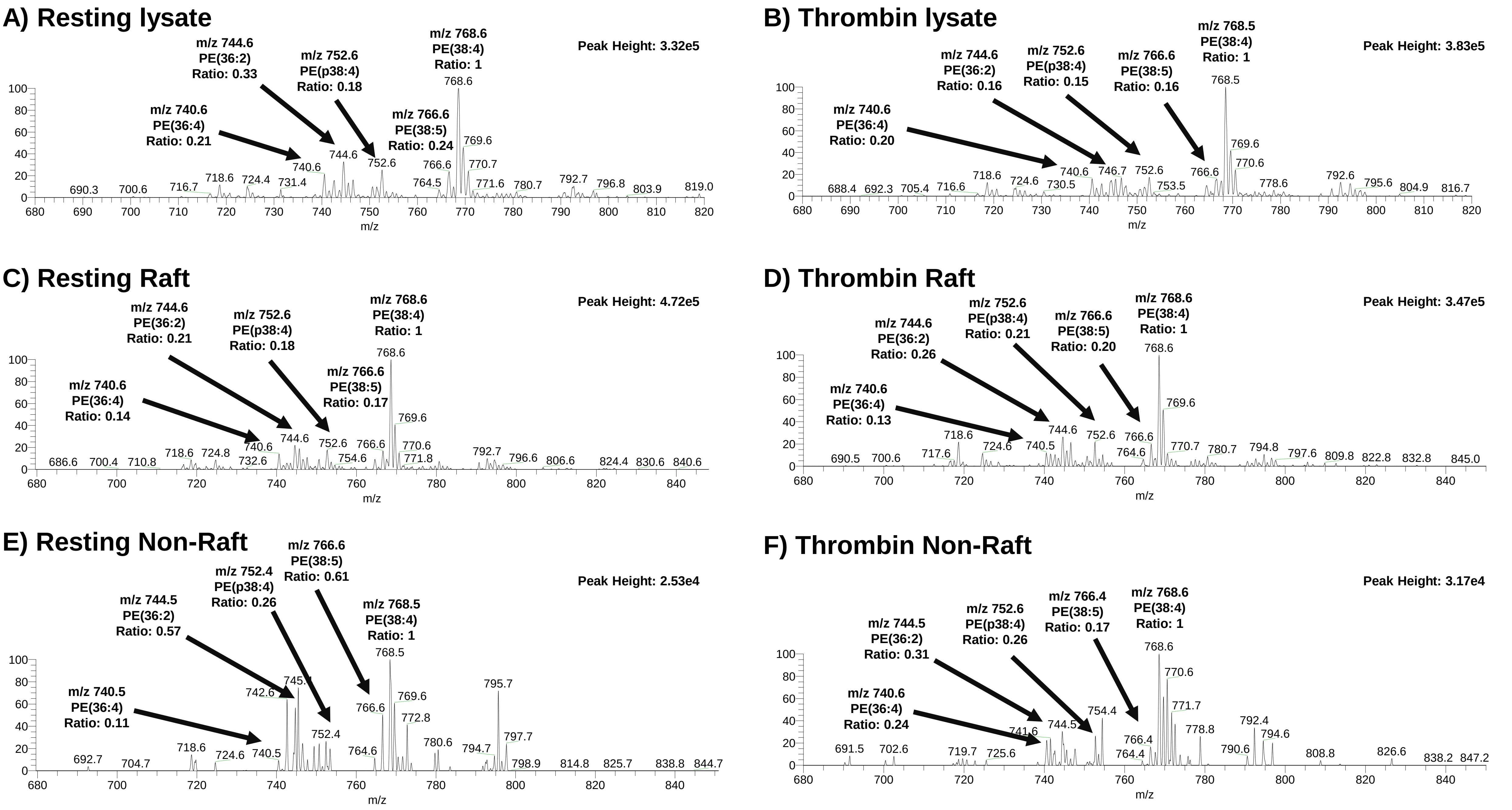

Fig. S4

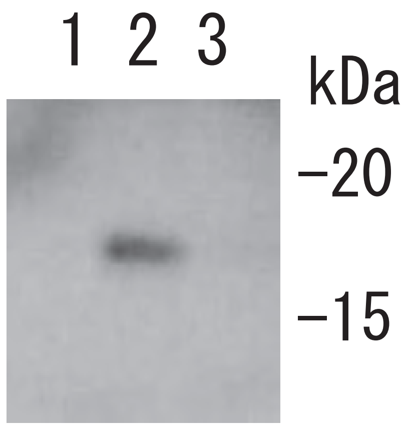

Figure S4 Phosphorylation of MLC(Ser19) in DRM rafts by thrombin.

Immunoblotting with anti-phospho-MLC (Ser19)antibody.

1 Resting platelets, 2 0.2U/ml Thrombin for 5 min,

3 10 μM Eptifibatide + 0.2U/ml Thrombin for 5 min.
